# Sacrificial layer-assisted nanoscale transfer printing

**DOI:** 10.1038/s41378-020-00195-1

**Published:** 2020-09-21

**Authors:** Junshan Liu, Bo Pang, Riye Xue, Rui Li, Jinlong Song, Xiaojun Zhao, Dazhi Wang, Xiaoguang Hu, Yao Lu, Liding Wang

**Affiliations:** 1 Key Laboratory for Micro/Nano Technology and System of Liaoning Province, Dalian University of Technology, Dalian, Liaoning 116024 China; 2 Key Laboratory for Precision and Non-Traditional Machining Technology of the Ministry of Education, Dalian University of Technology, Dalian, Liaoning 116024 China; 3 State Key Laboratory of Structural Analysis for Industrial Equipment, Department of Engineering Mechanics, Dalian University of Technology, Dalian, Liaoning 116024 China; 4 Department of Chemistry, School of Biological and Chemical Sciences, Queen Mary University of London, London, E1 4NS UK

**Keywords:** Nanoscience and technology, Nanoscale materials, Nanoscience and technology, Nanoscale materials

## Abstract

Transfer printing is an emerging assembly technique for flexible and stretchable electronics. Although a variety of transfer printing methods have been developed, transferring patterns with nanometer resolution remains challenging. We report a sacrificial layer-assisted nanoscale transfer printing method. A sacrificial layer is deposited on a donor substrate, and ink is prepared on and transferred with the sacrificial layer. Introducing the sacrificial layer into the transfer printing process eliminates the effect of the contact area on the energy release rate (ERR) and ensures that the ERR for the stamp/ink-sacrificial layer interface is greater than that for the sacrificial layer/donor interface even at a slow peel speed (5 mm s^−1^). Hence, large-area nanoscale patterns can be successfully transferred with a yield of 100%, such as Au nanoline arrays (100 nm thick, 4 mm long and 47 nm wide) fabricated by photolithography techniques and PZT nanowires (10 mm long and 63 nm wide) fabricated by electrohydrodynamic jet printing, using only a blank stamp and without the assistance of any interfacial chemistries. Moreover, the presence of the sacrificial layer also enables the ink to move close to the mechanical neutral plane of the multilayer peel-off sheet, remarkably decreasing the bending stress and obviating cracks or fractures in the ink during transfer printing.

## Nanofabrication: Nanoscale transfer printing using a sacrificial layer

Transfer printing is an emerging assembly technique for flexible and stretchable electronics, and a breakthrough method has been developed that allows transfer printing at the nanoscale by means of a weak-adhesion intermediate (sacrificial) layer. Although transfer printing has gained considerable attention for transferring various classes of materials with a wide range of geometries and configurations from one substrate to another, very few methods have allowed such printing at the nanoscale; those methods demand very elaborate processing. However, a team headed by Junshan Liu at Dalian University of Technology, China developed a simple, versatile transfer printing technique utilizing a sacrificial layer that for the first time permitted the successful transfer of large-area nanoscale patterns with a 100% yield. The authors intend to further investigate the possibilities of their innovative system and its application in various nanotechnologies.

## Introduction

Transfer printing is capable of transferring various classes of materials with a wide range of geometries and configurations (referred to as ink) from one substrate (referred to as the donor) to another via a stamp and has been extensively used for flexible and stretchable electronics^1^, such as bendable transistors^2^, stretchable nanophotonic devices^3^, stretchable radio frequency identification tags^4^, and flexible photodetectors^5^. While a variety of transfer printing methods have been developed^6–21^, the resolution of most of these methods, that is, the narrowest continuous line that can be reliably transferred, is at the micrometer scale. There are a few methods that allow the transfer of nanoscale patterns^19,20^. However, such methods require stamps with elaborately designed relief features and specially designed interfacial chemistries at the stamp-ink and ink-substrate interfaces for transferring specific materials; thus far, only metal patterns have been demonstrated^19,20,22–25^. In addition, the thickness of the metal patterns is limited to sizes smaller than 50 nm^22,23^.

Transfer printing is viewed as the competing fracture of the stamp/ink interface and the ink/substrate interface and is determined by the energy release rates (ERRs) for these two interfaces (G_stamp/ink_ and G_ink/substrate_)^26^. Kinetically controlling G_stamp/ink_ by controlling the peel speed of the stamp from the ink can enable transfer printing to be easily conducted and has been widely exploited^27–30^. However, to our best knowledge, this speed-based transfer printing method has never been used for transferring nanoscale patterns. This is mostly because the ERR is also related to the contact area in addition to the peel speed^26,31^. The contact area at the stamp/ink interface is generally considered to be equal to that at the ink/donor interface because it is believed that the flexibility of the stamp can help obtain a conformal contact^27^, so the effect of the contact area on the ERR is usually neglected. However, we believe that with a decrease of the feature size of the ink, the stamp can only partially contact the ink, and the difference in the contact areas between the stamp/ink interface and the ink/donor interface will increase until G_ink/substrate_ is much larger than G_stamp/ink_ and the adhesion between the stamp and the ink is not sufficiently large to pick up the ink, even at an extremely high peel speed. This also explains why Au patterns with feature sizes smaller than 100 μm cannot be peeled off from a donor substrate by 3 M Scotch tape in the “dry-taping” approach developed by Bao’s group^6^.

Here, we present a sacrificial layer-assisted nanoscale transfer printing method. A sacrificial layer was introduced into the transfer printing process to eliminate the effect of the contact area; hence, for the first time, large-area nanoscale patterns, such as 100-nm-thick, 4-mm-long, and 47-nm-wide Au nanoline arrays and 10-mm-long and 63-nm-wide lead zirconate titanate (PZT) nanowires, were successfully transferred using only a blank stamp and without the assistance of any interfacial chemistries. Moreover, the presence of the sacrificial layer enabled the ink to move close to the mechanical neutral plane of the multilayer peel-off sheet, remarkably decreasing the bending stress and obviating cracks or fractures in the ink^10^. The successful transfer of a variety of materials with diverse geometries and multilayer structures, such as metal-insulator-metal capacitors, demonstrates some of the capabilities and potential applications.

## Results and discussion

### Transfer printing process

**Fig. 1:**
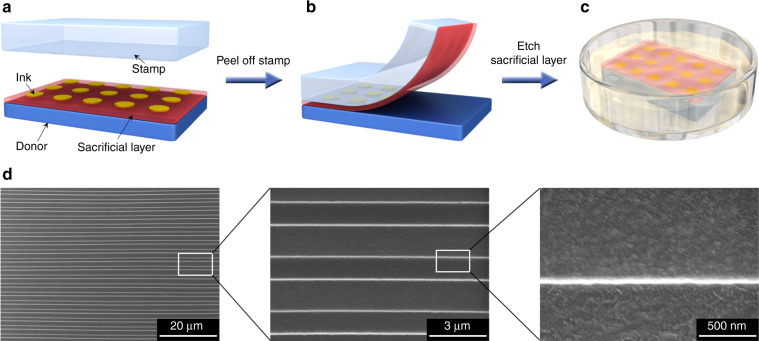
Nanoscale transfer printing. **a**–**c** Schematic illustration of the transfer printing process: deposit a sacrificial layer on a donor substrate, prepare ink on the sacrificial layer, and press a stamp on the surfaces of the ink and the sacrificial layer (**a**); peel off the stamp (**b**); and etch the sacrificial layer (**c**). **d** Images of 100-nm-thick, 4-mm-long, and 47-nm-wide Au nanoline arrays transferred onto tape

Figure 1a–c schematically illustrates the process of the sacrificial layer-assisted nanoscale transfer printing method. A sacrificial layer is deposited on the surface of a donor substrate, and ink is prepared on the sacrificial layer (Fig. 1a). Ink can be fabricated by a variety of methods, such as photolithography-based methods and electrohydrodynamic jet (e-jet) printing, so the classes and thicknesses of the ink are theoretically not limited. To facilitate separation, the sacrificial layer should have a weak adhesion to the donor substrate. The adhesion of deposited metal films on a polymethylmethacrylate (PMMA) plate is normally weak^32^; therefore, a Cu layer (70 nm) is used as the sacrificial layer and sputtered onto the surface of a PMMA donor substrate. Then, a stamp is pressed onto the surfaces of the ink and the sacrificial layer with an average pressure of 6.0 kPa. Here, 3 M Scotch tape (cat. 600) is used as the stamp for two reasons. First, 3 M Scotch tape has good adhesion to a variety of materials, which is beneficial for peeling the ink off the donor substrate. Therefore, it has been commonly used as the stamp in many kinds of transfer printing methods^6,14,33,34^. Second, 3 M Scotch tape is commercially available, low-cost, highly flexible, and transparent. Therefore, it is also one of the most popular substrates for flexible devices^33,35,36^. In fact, 3 M Scotch tape with transferred ink can itself be a flexible device in many cases, where the ink does not have to be transferred again from the 3 M Scotch tape to another substrate. The stamp is then peeled off (Fig. 1b). Differing from previously reported methods, the sacrificial layer is directly fabricated on the donor substrate and dominates the contact with the stamp in our method. Therefore, the sacrificial layer actually plays the role of the ink, and the ink is just sandwiched between the sacrificial layer and the stamp. Compared to the ink, the sacrificial layer is a continuous film, and its feature size is considered to be infinite; thus, the effect of the contact area on the ERR can be neglected. Moreover, the sacrificial layer has weak adhesion to the donor substrate. Therefore, the ERR for the stamp/ink-sacrificial layer interface is ensured to be larger than that for the sacrificial layer/donor interface even at a slow peel speed. In all the following experiments and discussions, unless otherwise specified, the sacrificial layer and the ink above it were picked up and transferred onto the tape at a peel speed of 5 mm s^−1^, which is much slower than the speed used in other studies^11,27^. This slower peel speed makes the operation easier to control and is suitable for wider classes of inks and substrates. The sacrificial layer is removed via chemical etching (Fig. 1c). For example, Cu can be etched by a mixture of H_4_CeN_2_O_3_, HClO_4_, and H_2_O (10 g:9 mL:100 mL), and then, the stamp with the transferred ink is rinsed with deionized water and blow-dried by nitrogen.

Figure 1d shows a 100-nm-thick Au nanoline array transferred onto 3 M Scotch tape (19 mm × 40 mm). This array contains 99 Au nanolines with a width of 47 ± 3 nm and a length of 4 mm and was originally fabricated on a PMMA donor substrate (30 mm × 30 mm × 2 mm) with a 70-nm-thick Cu sacrificial layer based on the edge lithography technique (Supplementary Fig. 1). As expected, Au nanolines were successfully transferred and remained intact with a yield of 100%.

**Fig. 2:**
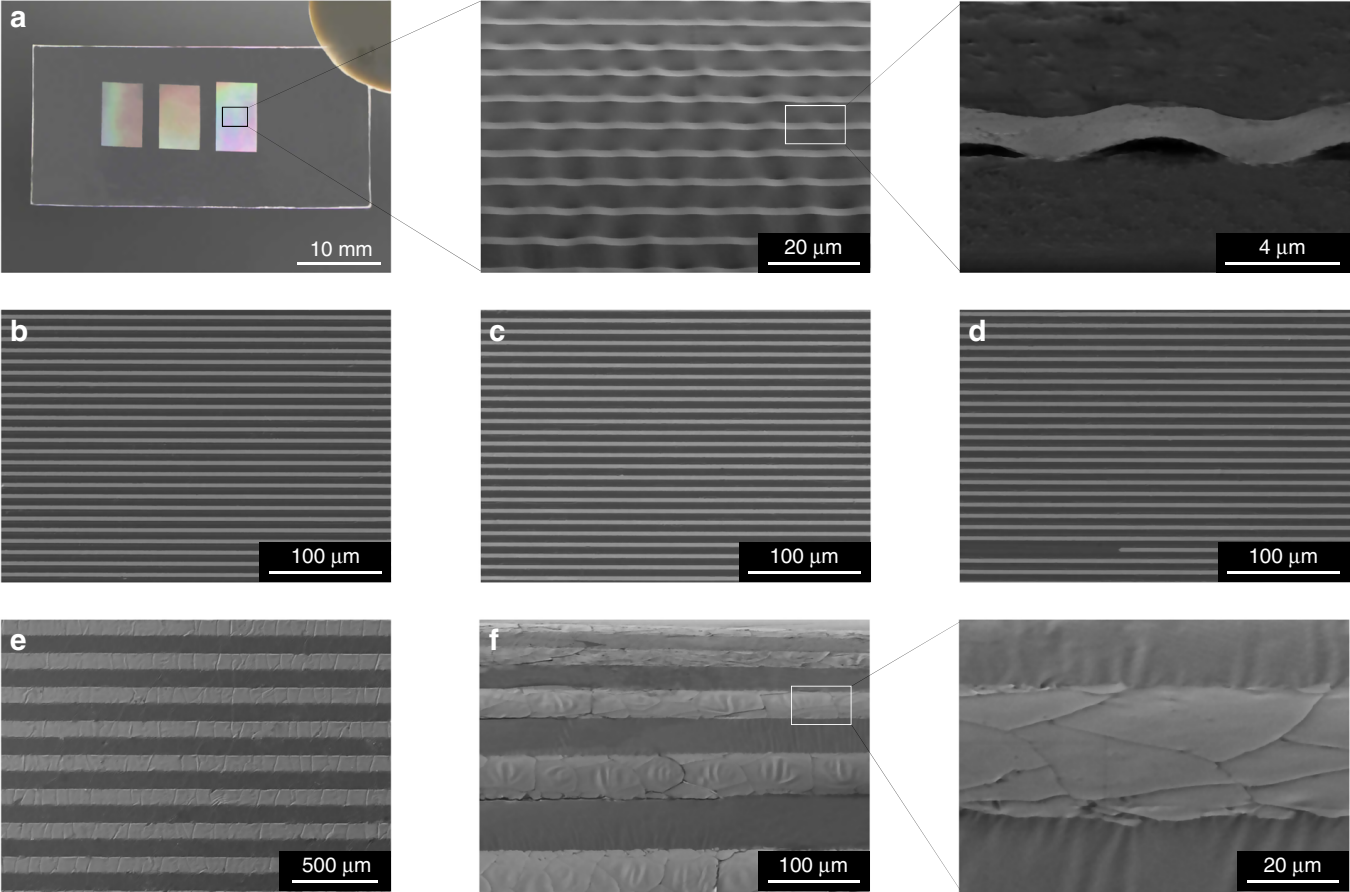
SEM and optical images of 100-nm-thick Au patterns. **a** 3-μm-wide Au line arrays transferred onto tape with the aid of a Cu sacrificial layer; **b**–**d** 3-μm-wide Au line arrays directly fabricated on a PMMA substrate (**b**) and after pressing tape and peeling it away at a speed of 5 mm s^−1^ (**c**) or at a speed of 15 mm s^−1^ (**d**). **e**, **f** SEM images of 100-μm-wide Au line arrays transferred onto tape without the aid of a sacrificial layer (**e**) and viewed at a 45° tilt angle (**f**)

To observe the interface between Au nanolines and the tape and check whether a conformal contact was achieved, we tilted the specimen at different angles in the scanning electron microscope (SEM); however, the interface was not clearly distinguishable because the Au lines are too narrow, and the tape has poor electrical conductivity (Supplementary Fig. 2). Therefore, we increased the width of the Au lines. Figure 2a shows three groups of 3-μm-wide Au line arrays (1600 Au lines in total) transferred onto tape. The 45° tilt view SEM images clearly show that the tape was only in partial contact with the Au lines. Therefore, if there is no Cu sacrificial layer, then G_stamp/ink_ would be much smaller than G_ink/substrate_ since the contact area at the tape-Au line interface is much smaller than that at the Au line-PMMA donor substrate interface, and 3-μm-wide Au lines would not be picked up by the tape regardless of how fast the peel speed is. To verify this inference, we fabricated the same 3-μm-wide Au line arrays directly on a PMMA substrate (Fig. 2b). Indeed, the 3-μm-wide Au lines could not be picked up at a peel speed of 5 mm s^−1^ (Fig. 2c and Supplementary Video 1), and only small portions of the Au lines were occasionally lifted off even at a fast speed of 15 mm s^−1^ (Fig. 2d and Supplementary Video 2). Nevertheless, when the width of Au lines directly fabricated on a PMMA substrate was increased to 100 μm, they could be easily lifted off at a slow peel speed of 5 mm s^−1^ (Fig. 2e and Supplementary Video 3), and the 45° tilt view SEM images show that the tape had an approximately conformal contact with the 100-μm-wide Au lines (Fig. 2f). Hence, it can be seen that the contact area does influence the ERR and thus the transfer printing process, and the contact area plays a more important role than the peel speed when the feature size of the ink is smaller than a critical value; the substantial improvement in the resolution of the transfer printing method reported here is indeed attributed to the presence of the sacrificial layer.

### Versatility of transfer printing

The method presented here can essentially transfer almost any class of materials as long as we properly select the sacrificial layer and the donor substrate. On the one hand, the fabrication techniques of the ink should be compatible with the sacrificial layer and the donor substrate. On the other hand, the sacrificial layer should have a weak adhesion to the donor substrate to facilitate separation. Silicon is compatible with a variety of micro- and nanofabrication techniques, including high-temperature processes that are not compatible with polymers, and it has poor adhesion to weakly reactive metals such as Cu, Au or Pt. Therefore, a silicon wafer as the donor substrate and a Cu or Au film as the sacrificial layer were selected as another example to examine the versatility and application potential of this method.

**Fig. 3:**
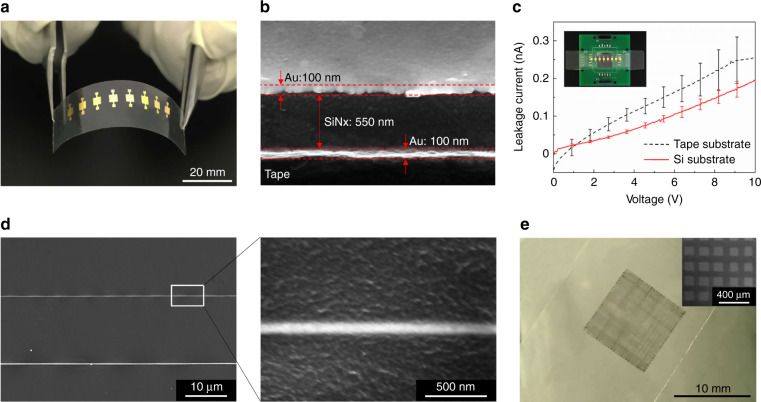
Micro- and nanostructures transferred from silicon donor substrates. **a** Eight capacitors transferred onto tape. **b** SEM image of the cross section of a capacitor viewed at a 45° tilt angle. **c** Current–voltage characteristics of capacitors. **d** PZT nanowires 10 mm long and 63 nm wide transferred onto tape. **e** Graphene patterns transferred onto tape

Figure 3a shows eight capacitors on tape transferred from a silicon donor substrate with a Cu sacrificial layer (70 nm). Each capacitor is composed of a 100-nm-thick bottom Au layer, a 550-nm-thick SiNx insulating layer, and a 100-nm-thick top Au layer. The SEM image (Fig. 3b) taken at a 45° tilt angle shows the cross section of the three-layer structure, which was first cut on the silicon wafer by a diamond pen and then transferred onto the tape. The SiN_x_ layer was deposited at 250 °C by plasma-enhanced chemical vapor deposition (PECVD). This deposition temperature is much higher than the glass transition temperature of PMMA (105 °C), so a PMMA plate could not be used as the donor substrate. All eight capacitors retained their functions after the transfer, and the performance of the capacitors transferred onto the tape was similar to that of the capacitors on the silicon wafer, as shown in Fig. 3c. This experiment also illustrates the ability of our method to transfer complex multilayer structures.

E-jet printing is a high-resolution additive manufacturing technique that can directly pattern classes of materials^37–39^. PZT nanowires were first e-jet-printed on a silicon wafer with a Au sacrificial layer (70 nm) and sintered at 600 °C in a muffle furnace. Here, we used a Au layer to replace the Cu layer as the sacrificial layer because Au has higher temperature stability. Then, the Au layer with PZT nanowires was peeled off from the silicon donor substrate by tape, and it was finally removed via chemical etching by a mixture of I_2_, KI, and H_2_O (1 g:5 g:50 mL). As shown in Fig. 3d, 10-mm-long and 63 ± 7-nm-wide PZT nanowires were successfully transferred onto the tape, which further demonstrates the nanoscale resolution of our method. With silicon donor substrates and a Au sacrificial layer, e-jet-printed graphene patterns were also successfully transferred onto tape (Fig. 3e).

**Fig. 4:**
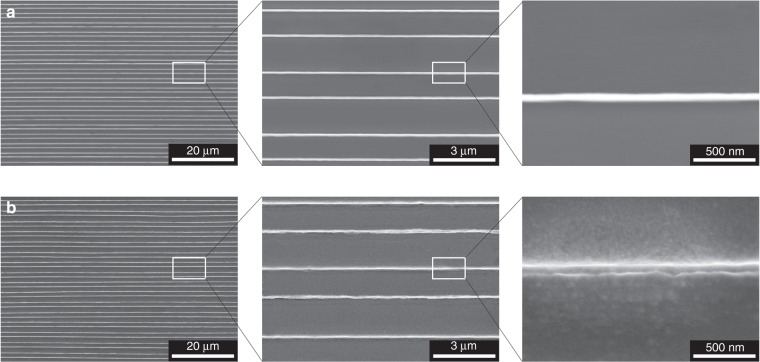
SEM images of Au nanoline arrays. Au nanoline array fabricated on a PMMA donor substrate (**a**) and transferred onto a PI receiver substrate by a PDMS stamp (**b**)

Furthermore, the lack of requirements for the interfacial chemistries or special relief features over the stamp suggests that the method reported here is suitable for a wide range of other stamps besides 3 M Scotch tape. For example, we used a blank polydimethylsiloxane (PDMS) plate as a stamp and transferred the same structured Au nanolines as shown in Fig. 1d from a PMMA donor substrate to a polyimide (PI) receiver substrate. Similarly, 99 Au nanolines (47 nm wide and 4 mm long) were originally fabricated on a PMMA donor substrate (30 mm × 30 mm × 2 mm) (Fig. 4a), and a blank PDMS stamp (20 mm × 40 mm × 1 mm) was pressed on the surfaces of the Au nanolines and the Cu sacrificial layer with an average pressure of 6.0 kPa. The PDMS stamp was peeled off from the PMMA donor substrate at a speed of 5 mm s^−1^, and the Cu sacrificial layer was etched away. The PDMS stamp was pressed on the surface of a PI film (30 mm × 30 mm × 75 μm) (Kapton HN, DuPont, USA) with the same pressure (6.0 kPa). It has been demonstrated that the adhesion between the stamp and the ink is speed sensitive because of the viscoelastic behavior of the stamp, and the adhesion decreases with decreasing separation speed^26,27^. Therefore, to transfer the Au nanowires from the PDMS stamp onto the PI film, the PDMS stamp was peeled off from the PI film at a slow separation speed of 5 mm s^−1^. As shown in Fig. 4b, Au nanolines were successfully transferred onto the PI receiver substrate with a yield of 100%.

### Effects of the sacrificial layer on the bending stress

**Fig. 5:**
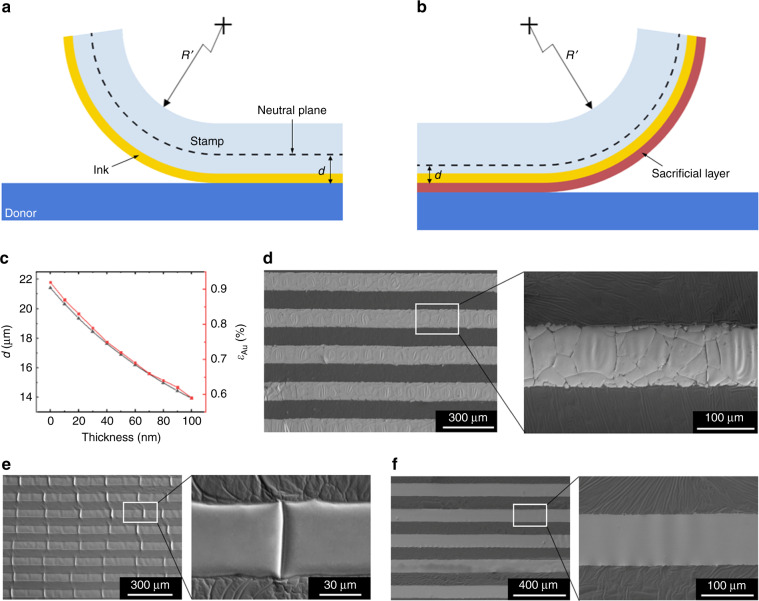
Effects of the sacrificial layer on the bending stress. **a**, **b** Diagrams of multilayer peel-off sheets without (**a**) or with (**b**) a sacrificial layer. The distance between the mechanical neutral plane and ink and the bending curvature radius of the stamp at the advancing edge of the separation region are denoted as *d* and *R’*, respectively. **c** Effects of the thickness of the Cu sacrificial layer on the distance (*d*) and the tensile strain (*ε*_Au_). **d**–**f** Images of Au line arrays transferred onto Scotch tape without the aid of a Cu sacrificial layer (**d**) or with the aid of a Cu sacrificial layer with a thickness of 10 nm (**e**) or 70 nm (**f**)

The ink inevitably endures a bending stress when the stamp is peeled off from the donor substrate. When the stress reaches the failure strength, cracks or even fractures will be induced in the ink^10^. In mechanics, there is a conceptual plane inside a sheet known as the mechanical neutral plane, where the material of the sheet is under no stress during bending. The farther the material is from the mechanical neutral plane, the larger the imposed bending stress is. The presence of the sacrificial layer changed the position of the mechanical neutral plane of the multilayer peel-off sheet and enabled the ink to move close to the mechanical neutral plane (Fig. 5a, b), thus decreasing the bending stress and avoiding cracks or fractures in the ink.

**Fig. 6:**
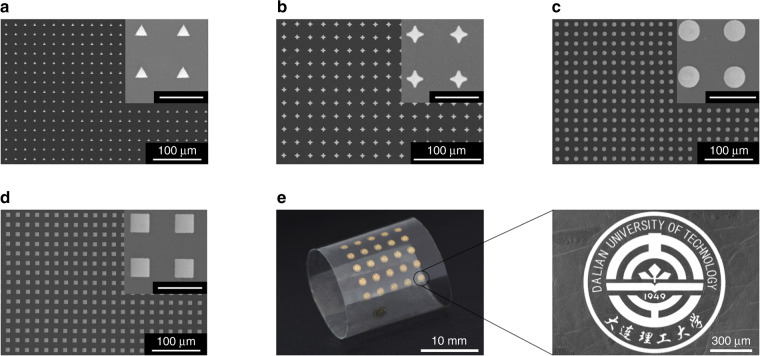
Images of Au patterns with different shapes and sizes transferred onto tape with the aid of a 70-nm-thick Cu sacrificial layer. The insets of (**a**–**d**) are enlarged SEM images (scale: 20 μm)

To analyze the effects of the sacrificial layer on the bending stress quantitatively, the multilayer peel-off sheet was studied based on composite beam theory in this work. For the Au-Scotch tape peel-off sheet, where the thickness of the Au layer was constant at 100 nm and the bending curvature radius of the tape at the advancing edge of the peel region (*R’*) was constant at 2.3 mm, the detailed theoretical calculation can be seen in Supplementary Note 1. When there was no sacrificial layer, the distance between the bottom of the Au film and the mechanical neutral plane of the peel-off sheet (*d*) was 24.19 μm, and the tensile strain in the Au film (*ε*_Au_) caused by the bending force was up to 1.04%. When there was a Cu sacrificial layer and a 10-nm-thick Cr protection layer, the distance between the bottom of the Au film and the mechanical neutral plane (*d*) was

1 $$d = \left( {1041357.1 - 130000h_1^2 - 5390h_1} \right)/\left( {48630 + 260000h_1} \right)$$

and the tensile strain (*ε*_Au_) in the Au film was

2 $$\varepsilon _{{\mathrm{Au}}} = \left( {1041357.1 - 130000h_1^2 - 5390h_1} \right)/\left( {113633045.9 + 130000h_1^2 + 613111390h_1} \right)$$

where *h*_1_ denotes the thickness of the Cu sacrificial layer, with units of microns. According to equations (1) and (2), the effects of the thickness of the Cu sacrificial layer on the distance between the bottom of the Au film and the mechanical neutral plane and on the tensile strain in the Au film are shown in Fig. 5c. It can be seen that with an increase in the thickness of the Cu sacrificial layer, the distance between the bottom of the Au film and the mechanical neutral plane decreased, and therefore, the tensile strain in the Au film correspondingly decreased. For example, when the thickness of the Cu sacrificial layer was 10 nm, *d* was 20.32 μm, and *ε*_Au_ was 0.86%; when this thickness was 100 nm, *d* was decreased to 13.92 μm, and *ε*_Au_ was decreased to 0.59%. The fracture strain of free-standing metal films is typically approximately 1%^40,41^. Therefore, the fracture of the Au lines can be obviated by optimizing the thickness of the Cu sacrificial layer. Figure 5d shows Au lines transferred without the assistance of a sacrificial layer, and many tiny cracks or even fractures were observed in the Au lines. When the thickness of the Cu sacrificial layer was 10 nm, regular cracks were still generated in the transferred Au lines (Fig. 5e). However, when the thickness of the Cu sacrificial layer was increased to 70 nm, *ε*_Au_ was 0.66%, the transferred Au lines had a smooth surface, and no cracks were observed (Fig. 5f). With the aid of a 70-nm-thick Cu sacrificial layer, Au patterns with a wide range of shapes and sizes were transferred onto Scotch tape, and the magnified SEM images in the insets show that no damage was caused to these Au patterns (Fig. 6a–e).

**Fig. 7:**
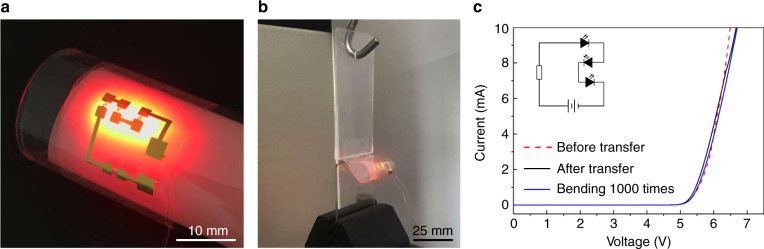
LED circuits transferred onto tape. **a** LED circuit bent around a glass tube. **b** Bending test of LED circuits. **c** Current–voltage characteristics of LED circuits

Nevertheless, we were surprised, though pleased, to find that the ink could sustain a large bending strain once it was transferred onto the stamp. For example, we manually bent 100-μm-wide Au lines transferred onto tape around a stainless tube with a 0.5 mm radius of curvature, and no cracks or fractures were observed in the Au lines, where the tensile strain in the Au film reached 4.53%, which is much larger than the fracture strain of Au films (~1%)^41^. Sustaining a large mechanical deformation is of great importance for flexible and stretchable electronics^42–44^. As a demonstration, we fabricated a simple flexible circuit, and this circuit was composed of transferred Au connectors, three embedded light-emitting diodes (LEDs), and one embedded resistor (Fig. 7a). After 1000 cycles of almost 180° bending and releasing (Fig. 7b), the Au connectors showed no cracking, and the performance of the circuit showed no obvious changes (Fig. 7c).

## Materials and methods

### Fabrication of microscale Au patterns

A 70-nm-thick Cu sacrificial layer was sputtered on the surface of a 2-mm-thick blank PMMA plate purchased from Goodfellow Cambridge Limited (Huntingdon, UK) at a sputtering power of 300 W. To protect the Cu layer from being etched by the etchant for Au, a 10-nm-thick Cr protection layer was then sputtered on the Cu layer at a sputtering power of 150 W. A 100-nm-thick Au layer was sputtered on the Cr layer at a sputtering power of 150 W. A positive photoresist (AZ MIR-703) was patterned on the surface of the Au layer. To avoid thermal deformation of the PMMA plate, the photoresist was soft-baked and hard-baked at a low temperature of 60 °C. The exposed gold was etched in a mixture of I_2_, KI and H_2_O (1 g:5 g:50 mL). The residual photoresist underwent a second exposure to UV light for 3 min without a photomask and was removed by the AZ 300 MIF developer.

### Transfer

3 M Scotch tape (cat. 600) was pressed onto the surfaces of the ink and the sacrificial layer by a finger with an average pressure of 6.0 kPa ± 1.3 kPa and then cut to the required dimensions with scissors. The tape was manually peeled off at a peel speed of 5 mm s^−1^. The tape was immersed into the etchant to remove the sacrificial layer. For the Cu sacrificial layer, the etchant was a mixture of H_4_CeN_2_O_3_, HClO_4_, and H_2_O (10 g:9 mL:100 mL), and the Cr protection layer was simultaneously removed by this etchant. For the Au sacrificial layer, the etchant was a mixture of I_2_, KI and H_2_O (1 g:5 g:50 mL). The tape was rinsed with deionized water and blow-dried by nitrogen.

### Fabrication of capacitors

A 70-nm-thick Cu sacrificial layer was sputtered on the surface of a 2-inch silicon wafer. A 10-nm-thick Cr protection layer was then sputtered on the Cu layer. A 100-nm-thick Au layer was sputtered on the Cr layer and then etched to form the top electrode of the capacitor. A 550-nm-thick SiN_x_ layer was deposited at 250 °C for 25 min by PECVD and etched by buffered hydrofluoric acid to form the insulating layer of the capacitor. A positive photoresist (BP212, Beijing Institute of Chemical Reagents, China) was spin-coated and patterned to protect the exposed solder pad of the top electrode from being etched by the etchant for the second Au layer. A second 100-nm-thick Au layer was sputtered and etched to form the bottom electrode of the capacitor. The residual photoresist was removed by acetone.

### E-jet printing

PZT nanowires were e-jet printed by a coaxial focused electrohydrodynamic jet (CFEJ) printing technique^39^. The homemade equipment for CFEJ printing comprised a computer-controlled three-dimensional movement stage, a coaxial needle unit, two syringe pumps, a high voltage power supply, and a microscopic vision system. The coaxial needle unit was composed of a 130-μm-diameter inner needle and a 1000-μm-diameter outer needle, and the inner needle protruded from the outer needle by 200 μm. The power supply was connected to the coaxial needle unit to provide an electric field. The two pumps were connected to the inlets of the inner and outer needles to provide the hydrodynamic force. A PZT sol and a high viscosity solution of silicone oil were delivered through the inner and outer needles, respectively, to form a stable coaxial jet. The silicone oil was used mainly because it was immiscible with the PZT sol and highly insulated and could be easily removed. Meanwhile, the silicone oil could protect the PZT sol from environmental disturbances, such as airflow, vibration, and temperature. When an electrical shearing force, the electric field-induced high viscous shearing force, and the internal pressure from the silicone oil solution were jointly applied on the PZT sol, the size of the PZT sol jet could be significantly decreased and remained stable at the nanometer scale. The distance between the inner needle and a silicon donor substrate (10 mm × 10 mm) with a 70-nm-thick Au sacrificial layer was 2 mm, a 5.5 kV voltage was applied between them, and the PZT sol encapsulated within the silicone oil was printed on the silicon substrate. The silicon substrate was heated at 200 °C for 20 min to evaporate organic solvents and solidify the PZT sol and then immersed in analytical grade isopropanol to remove the silicone oil. Graphene patterns were e-jet printed following a previously reported method^38^. Briefly, ethyl cellulose-dispersed graphene ink was delivered to a 50-μm-diameter needle at a flow rate of 5 × 10^−11^ m^3^ s^−1^. The distance between the needle and a silicon substrate with a Au sacrificial layer was 300 μm. A 1.0 kV voltage was applied, and the graphene ink was printed on the silicon substrate. The silicon substrate was heated at 350 °C for 30 min to remove organic solvents.

## Conclusions

In summary, a simple nanoscale transfer printing method using a weak-adhesion sacrificial layer was developed. Introducing the sacrificial layer into the transfer printing process not only substantially enhances the resolution but also remarkably decreases the bending stress and obviates cracks or fractures in the ink. Its versatility was demonstrated by transferring classes of materials prepared either by top-down or bottom-up techniques. In the near future, the capabilities of the reported method for transferring multiscale structures or repeated transferring will be examined. In addition, applications of the method especially in nanotechnologies where large-area nanopatterning is required, such as surface-enhanced Raman scattering (SERS) and subwavelength optical elements, will be developed.

## Supplementary information

Supplementary Information

Supplementary Video 1

Supplementary Video 2

Supplementary Video 3
