# Supplementary material for: Sacrificial layer-assisted nanoscale transfer printing
Source: Microsyst Nanoeng. 2020 Sep 21;6:80. doi: 10.1038/s41378-020-00195-1 (PMC8433480; doi:10.1038/s41378-020-00195-1)
Supplement: Supplementary file 1 — Supplementary Information [file 41378_2020_195_MOESM1_ESM.docx]

**Supporting Information**

**Sacrificial layer-assisted nanoscale transfer printing**

Junshan Liu,^*a,b^ Bo Pang,^a^ Riye Xue,^c^ Rui Li,^c^ Jinlong Song,^a^ Xiaojun Zhao,^a^ Dazhi Wang,^a^ Xiaoguang Hu,^a^ Yao Lu^d^ and Liding Wang,^a,b^

^a^Key Laboratory for Micro/Nano Technology and System of Liaoning Province, Dalian University of Technology, Dalian, Liaoning, 116024, P.R. China.

^b^Key Laboratory for Precision and Non-traditional Machining Technology of Ministry of Education, Dalian University of Technology, Dalian, Liaoning, 116024, P.R. China,

^c^State Key Laboratory of Structural Analysis for Industrial Equipment, Department of Engineering Mechanics, Dalian University of Technology, Dalian, Liaoning, 116024, P.R. China.

^d^Department of Chemistry, School of Biological and Chemical Sciences, Queen Mary University of London, London E1 4NS, UK.

*E-mail: liujs@dlut.edu.cn

**
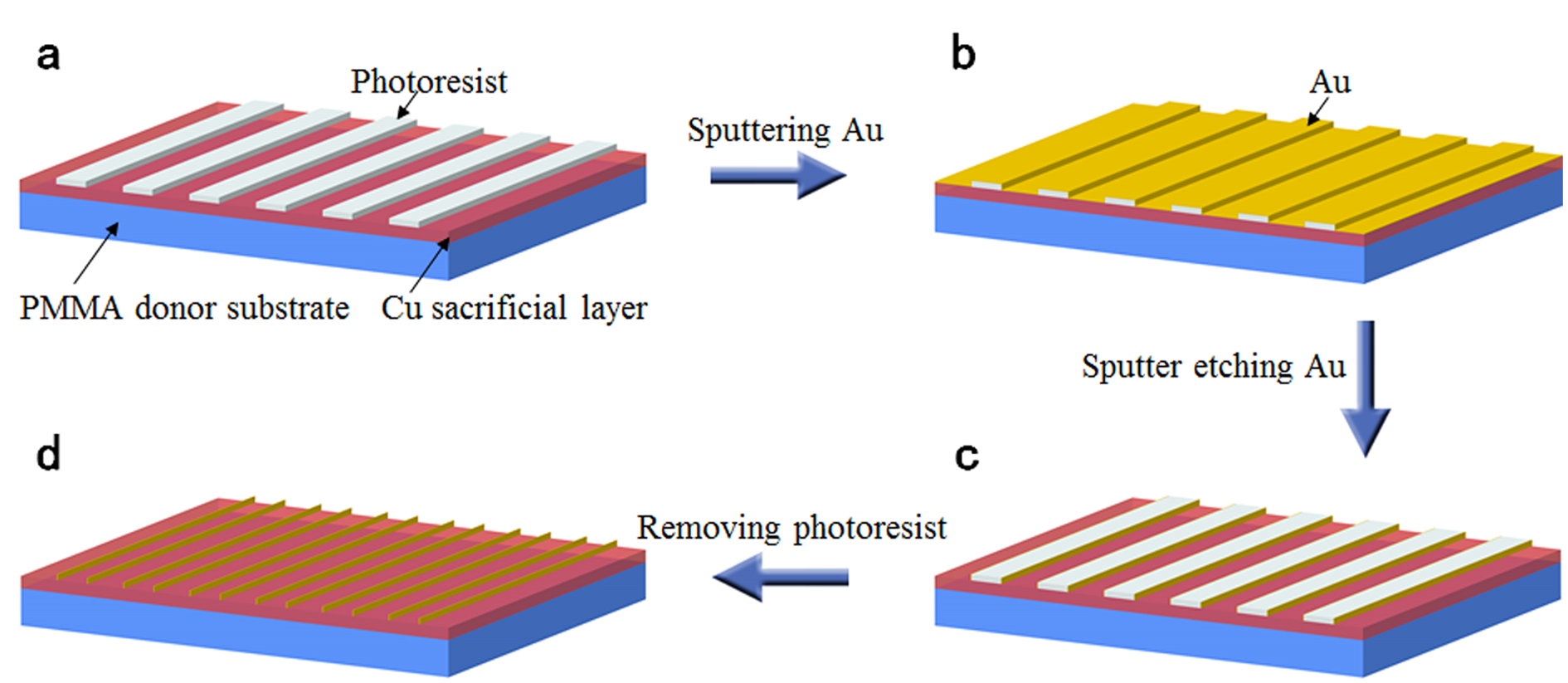
**

Fig. S1. Fabrication of nanoscale Au patterns on a PMMA donor substrate with a Cu sacrificial layer. (a) Patterning microscale photoresist mesas on the Cu sacrificial layer. (b) Sputtering an Au film on the surface of the photoresist mesas. (c) Sputter etching of the Au film on the horizontal surface of the photoresist mesas. (d) Removing the photoresist mesas by oxygen plasma to form nanoscale Au patterns.


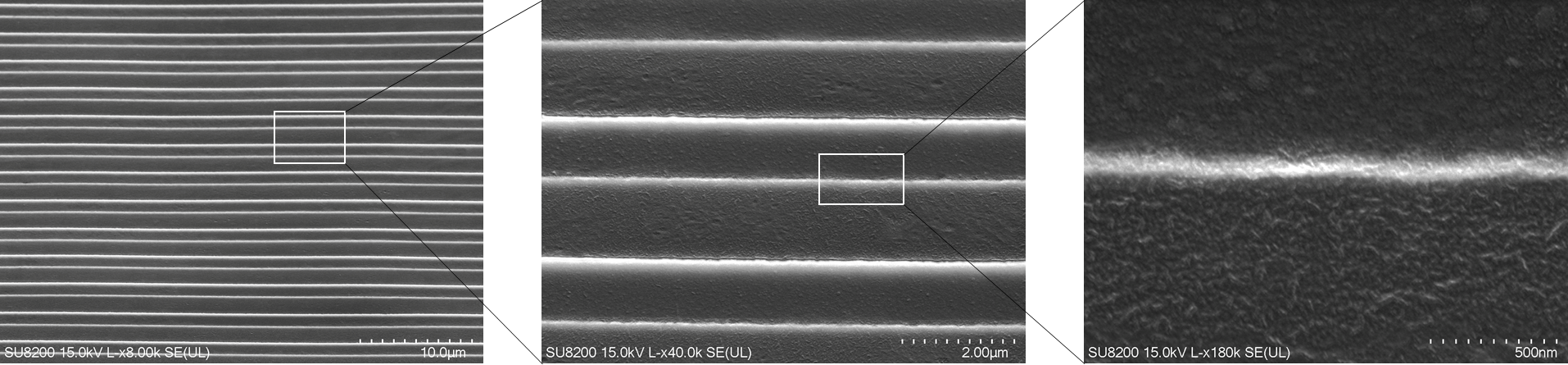


Fig. S2. SEM images of 100-nm-thick, 4-mm-long and 47-nm-wide Au nanoline arrays transferred on a tape viewed at 45° tilt angle. The interface between Au nanolines and the tape is not clearly distinguishable.

**Note S1:** Theoretical calculation of the tensile strain in the Au film.

**
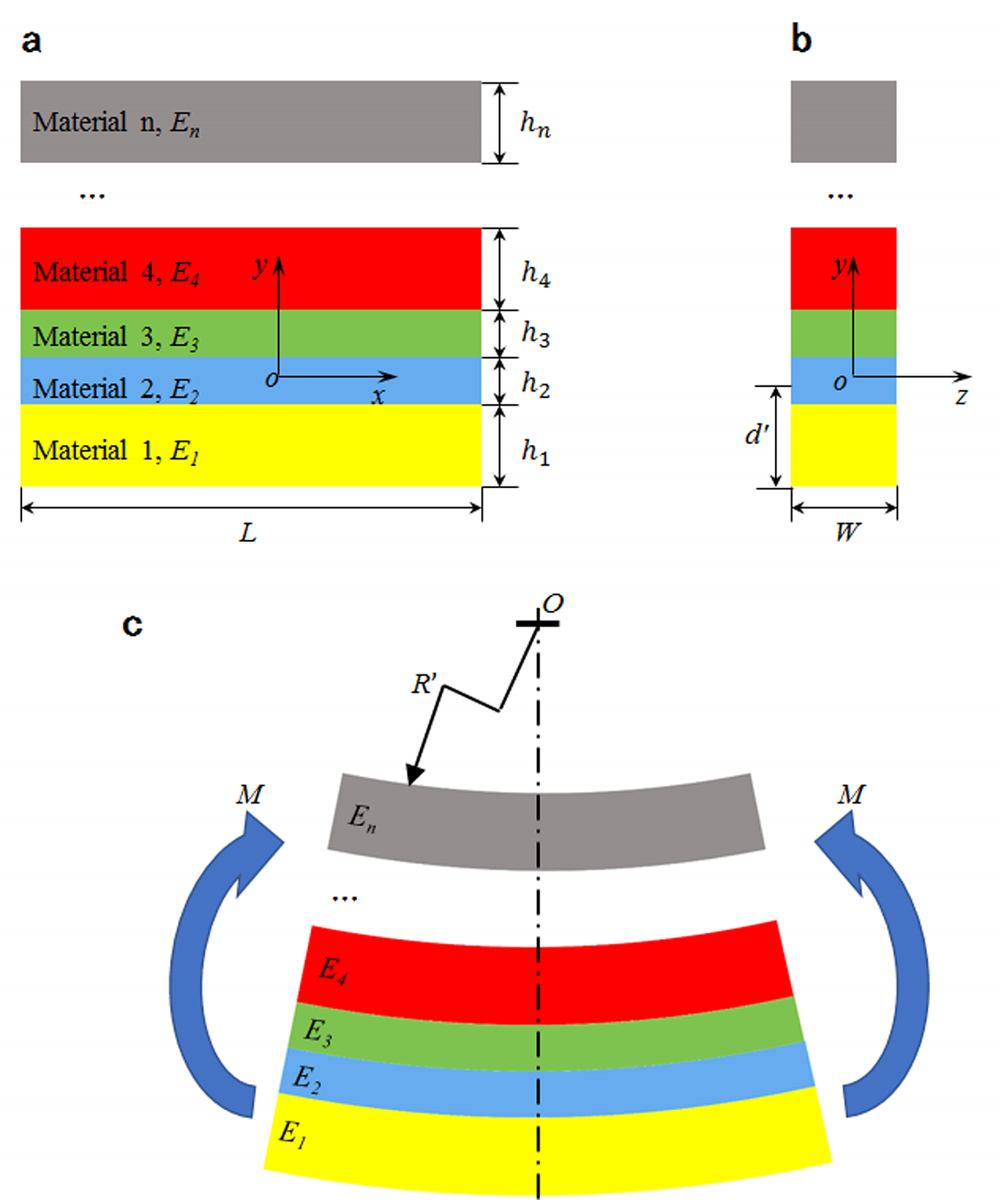
**

Fig. S3. Deformations of a composite beam in pure bending. (a) Side view of the composite beam. (b) Cross section of the composite beam. (c) The deformed composite beam subject to a bending moment (*M*).

In this work, the multi-layer peel-off sheet is studied based on a composite beam theory.^1^ As shown in Fig S3, a composite beam with a length of *L* and a width of *W* consists of *n*-layer materials, and the *i*th layer has a thickness of *h_i_* and a Young's modulus of *E_i_*. *R’* in Fig S3c denotes the bending curvature radius of the stamp at the advancing edge of the separation region when the stamp is peeled off from the donor substrate. We used a high-speed camera to capture the images of the peel-off progress, and then the images were converted to greyscale and used to measure *R’* using ImageJ software. As shown in Fig S4, when a piece of 3M Scotch tape (cat. 600) was peeled off from the PMMA donor substrate, the measured *R’* was equal to 2.3 mm.


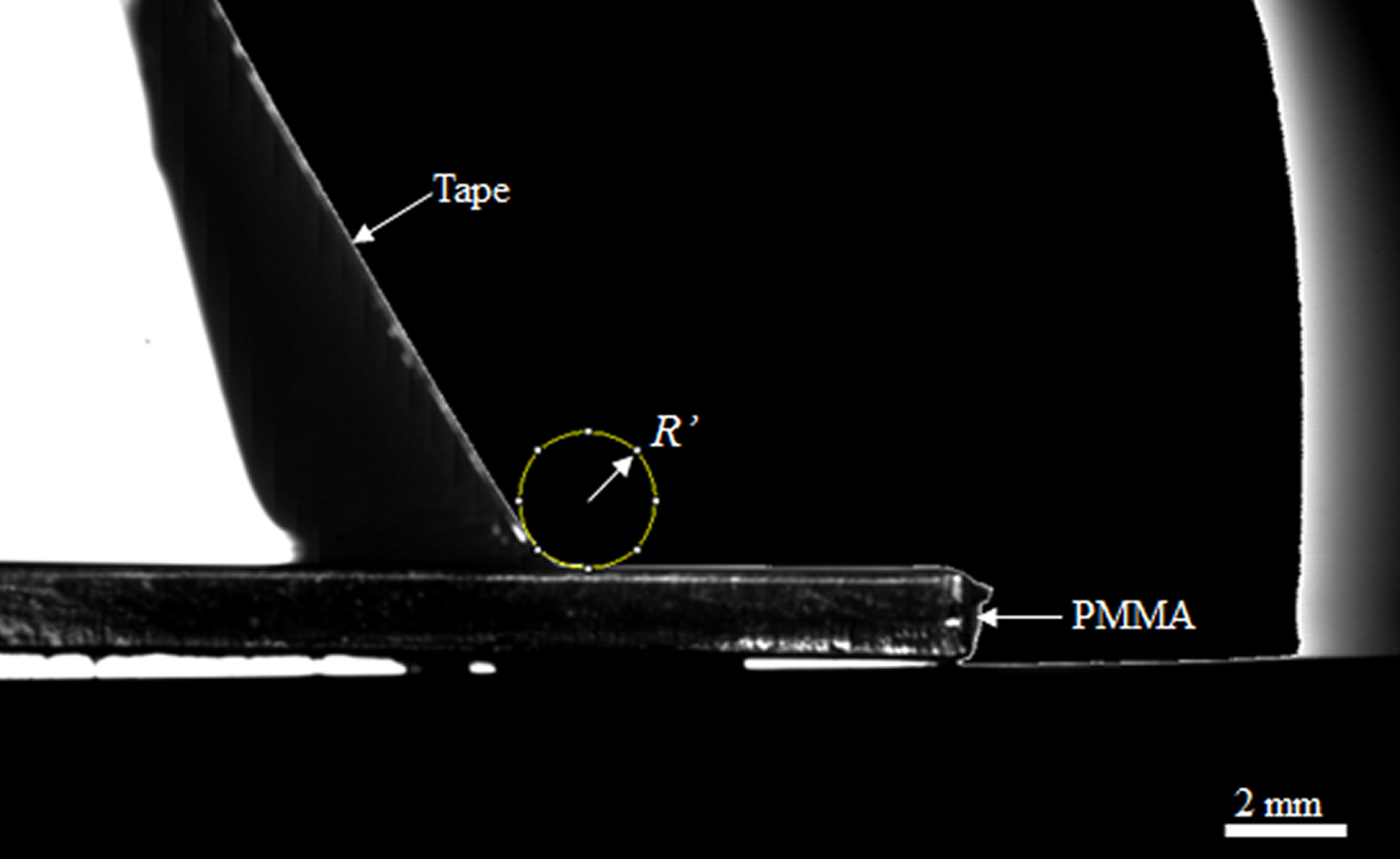


Fig. S4 Image of peeling off the tape from the PMMA donor substrate.

In Fig S3, the *xy* plane is symmetry, the *xz* plane is the mechanical neutral plane of the composite beam, *d’* denotes the distance from the neutral axis to the bottom of the composite beam, and all materials behave in a linearly elastic manner. The stress in the *i*th layer material ($\sigma_{xi}$) is

$$\sigma_{xi}=E_{i}\varepsilon_{i}=-\frac{E_{i}y}{R} (S1)$$

where $\varepsilon_{i}$ is the strain in the *i*th layer material, $y$ is the distance from the neutral axis, and *R* is the radius of curvature,

$$R=R^{'}+\sum_{i=1}^{n} h_{i}-d^{'}. (S2)$$

The resultant axial force acting on the cross section is equal to zero; hence,

$$\sum_{i=1}^{n} \int_{i} \sigma_{xi}dA=\sum_{i=1}^{n} \int_{i} -\frac{E_{i}y}{R}dA=0 (S3)$$

For the Au-tape peel-off sheet without the Cu sacrificial layer, the composite beam consists of an Au film ($E_{1}=82 GPa, h_{1}=100 nm$), an acrylic adhesive of the tape ($E_{2}=1.25 MPa, h_{2}=20 \mu m$), and a polypropylene backing of the tape ($E_{3}=350 MPa, h_{3}=38 \mu m$); thus we can obtain the following:

$$\int_{1} \sigma_{x1}dA+\int_{2} \sigma_{x2}dA+\int_{3} \sigma_{x3}dA=-\frac{E_{1}W}{R}\int_{-d'}^{-\left( d'-h_{1} \right)} ydy$$

$$-\frac{E_{2}W}{R}\int_{-\left( d^{'}-h_{1} \right)}^{\sum_{i=1}^{2} h_{i}-d^{'}} ydy-\frac{E_{3}W}{R}\int_{\sum_{i=1}^{2} h_{i}-d^{'}}^{\sum_{i=1}^{3} h_{i}-d^{'}} ydy=0 (S4)$$

Then, we can obtain *d’*:

$$d'=\frac{E_{1}h_{1}^{2}+2E_{2}h_{1}h_{2}+E_{2}h_{2}^{2}+2E_{3}h_{1}h_{3}+2E_{3}h_{2}h_{3}+E_{3}h_{3}^{2}}{2\left( E_{1}h_{1}+E_{2}h_{2}+E_{3}h_{3} \right)}=24.19 \mu m (S5)$$

Thus, we can obtain the distance between the Au film and the mechanical neutral plane (*d*). Here, *d* is equal to *d’*, which is 24.19 μm, because the Au film is just located at the bottom of the composite beam.

According to equation S2, we can obtain the radius of curvature (*R*):

$$R=R^{'}+\sum_{i=1}^{3} h_{i}-d^{'}=2333.91 \mu m$$

Therefore, the strain in the Au layer (*ε_Au_*) is

$$\varepsilon_{Au}=\varepsilon_{1}=\frac{d}{R}=\frac{24.19}{2333.91}=1.04\% (S6)$$

For the Au-tape peel-off sheet with the Cu sacrificial layer, the composite beam consists of a Cu sacrificial layer ($E_{1}=130 GPa$) with a thickness of $h_{1}$, a Cr protection layer ($E_{2}=279 GPa, h_{2}=10 nm$), an Au layer ($E_{3}=82 GPa, h_{3}=100 nm$), an acrylic adhesive of the tape ($E_{4}=1.25 MPa, h_{4}=20 \mu m$), and a polypropylene backing of the tape ($E_{5}=350 MPa, h_{5}=38 \mu m$). Similarly, we can obtain the following:

$$\int_{1} \sigma_{x1}dA+\int_{2} \sigma_{x2}dA+\int_{3} \sigma_{x3}dA+\int_{4} \sigma_{x4}dA+\int_{5} \sigma_{x5}dA$$

$$=-\frac{E_{1}W}{R}\int_{-d'}^{-\left( d'-h_{1} \right)} ydy-\frac{E_{2}W}{R}\int_{-\left( d'-h_{1} \right)}^{\sum_{i=1}^{2} h_{i}-d'} ydy-\frac{E_{3}W}{R}\int_{\sum_{i=1}^{2} h_{i}-d'}^{\sum_{i=1}^{3} h_{i}-d'} ydy$$

$$-\frac{E_{4}W}{R}\int_{\sum_{i=1}^{3} h_{i}-d'}^{\sum_{i=1}^{4} h_{i}-d'} ydy-\frac{E_{4}W}{R}\int_{\sum_{i=1}^{4} h_{i}-d'}^{\sum_{i=1}^{5} h_{i}-d'} ydy=0 (S7)$$

Then, we can obtain *d’*:

$$d'=\frac{E_{1}h_{1}^{2}+2E_{2}h_{1}h_{2}+E_{2}h_{2}^{2}+2E_{3}h_{1}h_{3}+2E_{3}h_{2}h_{3}+E_{3}h_{3}^{2}+2E_{4}h_{1}h_{4}+2E_{4}h_{2}h_{4}}{2\left( E_{1}h_{1}+E_{2}h_{2}+E_{3}h_{3}+E_{4}h_{4}+E_{5}h_{5} \right)}$$

$$+\frac{2E_{4}h_{3}h_{4}+E_{4}h_{4}^{2}+2E_{5}h_{1}h_{5}+2E_{5}h_{2}h_{5}+2E_{5}h_{3}h_{5}+2E_{5}h_{4}h_{5}+E_{5}h_{5}^{2}}{2\left( E_{1}h_{1}+E_{2}h_{2}+E_{3}h_{3}+E_{4}h_{4}+E_{5}h_{5} \right)}$$

$$=\frac{1041843.4+130000h_{1}^{2}+45840h_{1}}{48630+260000h_{1}} (S8)$$

In this case, the Au layer is the third layer of the composite beam, and the distance between the Au film and the mechanical neutral plane (*d*) is

$$d=d^{'}-h_{1}-h_{2}=\frac{1041357.1-130000h_{1}^{2}-5390h_{1}}{48630+260000h_{1}} (S9)$$

According to equation S2, we can obtain the radius of curvature (*R*):

$$R=R^{'}+\sum_{i=1}^{5} h_{i}-d^{'}$$

Therefore, the strain in the Au layer is

$$\varepsilon_{Au}=\varepsilon_{3}=\frac{d}{R}=\frac{1041357.1-130000h_{1}^{2}-5390h_{1}}{113633045.9+{130000h}_{1}^{2}+613111390h_{1}} (S10)$$

**Video Captions**

Video S1：3-μm-wide Au lines picked up at a peel speed of 5 mm s^-1^.

Video S2: 3-μm-wide Au lines picked up at a peel speed of 15 mm s^-1^.

Video S3: 100-μm-wide Au lines picked up at a peel speed of 5 mm s^-1^.

**Reference**

1. J. M. Gere and B. J. Goodno, *Mechanics of Materials*, Cengage Learning, Toronto, Canada, 2009, pp 458-459.
